# Predictors of Everyday Care Preference Importance Ratings for Veterans Living in the Nursing Home Setting

**DOI:** 10.1093/geroni/igaa057.300

**Published:** 2020-12-16

**Authors:** Caroline Madrigal, Lan Jiang, Whitney Mills, Wen-Chih Wu, Diane Berish, Tonya Roberts, Kimberly Van Haitsma, James Rudolph

**Affiliations:** 1 Providence VA Medical Center, Providence, Rhode Island, United States; 2 Pennsylvania State University, University Park, Pennsylvania, United States; 3 University of Wisconsin-Madison, Madison, Wisconsin, United States; 4 Penn State University, University Park, Pennsylvania, United States

## Abstract

Preference-based care is required by the Centers for Medicare and Medicaid Services and is linked to improved quality of nursing home care. The federally mandated Minimum Data Set (3.0) Preference Assessment Tool (PAT) is a 16-item standardized measure used to facilitate preference-based care through rating how important residents’ daily and activity preferences are. Little work has explored how Veterans’ unique demographic backgrounds and functional/cognitive abilities may influence how they rate their preferences (compared to general nursing home residents). Therefore, the purpose of this retrospective study was to explore the relationships between Veterans’ demographic/clinical characteristics and number of preference importance ratings. Our sample (n=194,068) consisted of Veterans admitted to community nursing homes after hospitalization at a Veterans Affairs facility for heart failure between 2010-2015. We used ordinal regression to explore predictors of preference importance ratings. Veterans were, on average, 78-years-old (SD=10.42) and mostly male (95%), white (81%), married (46%), cognitively intact (74%) with extensive functional impairment (60%) and minimal depressive symptoms (74%). Veterans rated an average of 12.47 preferences as important (SD=2.86; range=0-16). Veterans living with cognitive impairment, depression, and extensive functional impairment who were not married or separated had a lower number of important preferences (all p<0.0001). Veterans that were female, under the age of 85, and any race but white had a higher number of important preferences (all p<0.0001). Discussion will include implications for planning and delivering preference-based care for Veterans as well as next steps in research and practice to better understand and fulfill Veterans’ everyday care preferences.

